# Evaluating the TUITEK^®^ patient support program in supporting caregivers of children diagnosed with growth hormone deficiency in Argentina

**DOI:** 10.3389/fendo.2023.1129385

**Published:** 2023-04-06

**Authors:** Aria Reza Assefi, Selina Graham, María Lourdes Crespo, Matías Debicki, Jonathan Reston, Judit Gonzalez, Amrit Jheeta, Ekaterina Koledova

**Affiliations:** ^1^ Medical Department, Merck S.A. (an affiliate of Merck KGaA, Darmstadt, Germany), Buenos Aires, Argentina; ^2^ Atlantis Healthcare, London, United Kingdom; ^3^ Global Medical Affairs Cardiometabolic and Endocrinology, Merck KGaA, Darmstadt, Germany

**Keywords:** adherence, caregiver, electronic injection device, growth disorders, pediatric, patient support program, recombinant human growth hormone treatment, short stature

## Abstract

**Introduction:**

The appropriate use of recombinant human growth hormone (r-hGH) treatment provides an opportunity to improve growth outcomes among pediatric patients with growth hormone deficiency (GHD). However, a major challenge in clinical practice is to adequately recognize and address factors that negatively affect treatment adherence. TUITEK^®^ patient support program (PSP) was designed to help caregivers of children diagnosed with GHD to personalize the care pathway, improve adherence, and achieve better outcomes. Effectiveness of TUITEK^®^ PSP has been demonstrated previously in a smaller sample (n = 31) in Taiwanese population. Here, we present the results from Argentina.

**Methods:**

TUITEK^®^ PSP was piloted among 76 caregivers of children with GHD administering r-hGH using easypod™ (Merck KGaA, Darmstadt, Germany) auto-injector device in Argentina. Based on TUITEK^®^ personalization questionnaire, caregivers were assigned to high- and low-risk groups across four categories that may influence adherence, including disease and treatment coherence (DTC), self-administration (SA), treatment-related anxiety (TRA), and emotional burden (EB). The caregivers who were included in atleast one high-risk group had the provision of telephone calls with a nurse practitioner every 2 weeks for 3 months. The Wilcoxon signed-rank test was employed to assess changes in questionnaire-based scoring patterns between baseline and follow-up evaluations.

**Results:**

Statistically significant changes (*p* < 0.05) in questionnaire scores between baseline and follow-up evaluations were observed across the four categories. The mean/median DTC (n = 11) and SA (n = 23) scores changed from 2.45/3 and 2.17/2, respectively, to 4/4, with all the caregivers moving to low-risk group following program completion (100%) for both categories. The mean/median TRA score (n = 40) changed from 3.58/3 to 2.5/2 and 67.5% of patients (27/40) moved to low-risk group. The mean/median EB score (n = 32) changed from 3.69/3 to 3.13/3 however, none of the caregivers moved to low-risk group (0%).

**Conclusion:**

TUITEK^®^ PSP is a simple, practical, and time-efficient interventional tool that can be used to address key adherence-related issues among caregivers of children with GHD and provide personalized adherence support. Our findings demonstrate that TUITEK^®^ PSP has the potential to improve treatment adherence and self-management, thereby improving growth outcomes in Argentina.

## 1 Introduction

Growth hormone deficiency (GHD) is a rare, chronic condition characterized by the insufficient production of growth hormones during childhood, which affects the growth rate (1, 2). Recombinant human growth hormone (r-hGH) therapy is an established treatment for GHD administered *via* daily subcutaneous injections from early childhood to adolescence (1–5). Although r-hGH treatment considerably improves the height outcome potential of children and adolescents with short stature (1, 5–8), the treatment nonadherence rate remains high (9). Treatment adherence has been defined as “the extent to which a patient’s behavior matches agreed recommendations from their health professional” (2, 10). Several studies have examined the prevalence of nonadherence among children and adolescents with clinical indications for r-hGH treatment, including GHD, Turner syndrome, small for gestational age (SGA), and chronic renal failure (CRF), and reported that up to 71% and 82% of children and adolescents, respectively, were nonadherent to their treatment (1, 9). Poor adherence to the r-hGH treatment negatively affects both long-term clinical outcomes (leading to suboptimal growth outcomes) (9, 11–14) and healthcare system cost and resources (10, 15–18). Living with GHD can be both physically and emotionally challenging for the patient and their family (11, 19–22). Challenges may include insufficient knowledge regarding the disease and its treatment, discomfort and pain associated with daily injections, lack of self-efficacy, anxiety in administering treatment, or concerns about the long-term effects of treatment (9). Several studies have previously highlighted the negative impact of such risk factors on treatment adherence and the importance of identifying and addressing potentially modifiable factors, such as perceptions and beliefs, to improve clinical and behavioral outcomes of the care pathway (1, 12, 16, 19). However, a major challenge within clinical practice is to adequately recognize and manage the factors that negatively affect treatment adherence.

In this regard, the patient support program (PSP) help patients with GHD and their families to optimize the use of their prescribed r-hGH treatment and self-manage their long-term condition (23–28). Various PSP approaches have been previously successful in improving adherence to r-hGH treatment, including a targeted educational intervention supported by digital medication adherence monitoring (29) and a nurse-led personalized intervention (30). Specifically, the TUITEK^®^ PSP has been designed and developed to deliver personalized support tailored to the individual needs of patients, caregivers, and healthcare providers throughout the different phases of their treatment care pathway. This study aimed to evaluate the impact of the TUITEK^®^ PSP on the personal perceptions and beliefs of caregivers of children with GHD who underwent r-hGH treatment in Argentina.

## 2 Materials and methods

This prospective research was conducted to determine the impact of the TUITEK^®^ PSP. Briefly, the program was delivered by PSP nurse practitioners and comprised two key components: 1) a training session with the aim of developing key skills and strategies to effectively deliver the program, and 2) a PSP manual containing a personalization questionnaire directed to caregivers to identify the priority topics, scheduled telephone call guide, and resource packs incorporating behavior change techniques and motivational interviewing.

### 2.1 Participant recruitment and selection

**Figure 1:**
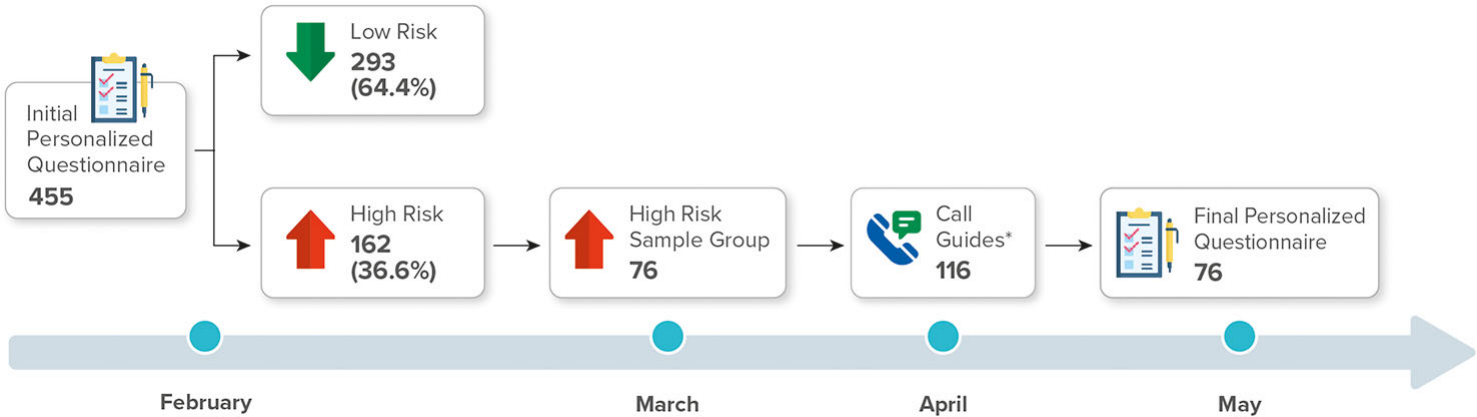
Flowchart of the participant recruitment process.

The TUITEK^®^ PSP was conducted among 76 caregivers of children with GHD who underwent treatment with r-hGH using easypod™ (Merck KGaA, Darmstadt, Germany) auto-injector device in Argentina for 3 months. The participants were recruited from 23 provinces in Argentina between February and May 2021. The caregivers (mother, father, or immediate caregiver) of children who were diagnosed with short stature due to GHD and who were undergoing r-hGH treatment were randomly selected (n = 455). The eligible participants completed a bespoke set of personalization questions, which explored their experiences, perceptions, and beliefs regarding their child’s condition and treatment. Risk assessment scores were obtained using a 5-point rating scale (**Supplementary Material Table S1**). The personalization questionnaire categorized individuals into low-risk (n = 293, 64.4%) and high-risk (n = 162, 35.6%) groups. From the high-risk group, 76 caregivers were randomized, using the excel program, who formed our sample. All caregivers consented to participate in the TUITEK^®^ program (**Figure 1**). Written informed consent was obtained from each eligible participant at the start of the program.

### 2.2 Data collection

Of the participating group, a score was calculated to identify ‘high-risk’ priority factor-based topics based on the responses collected in each caregiver’s baseline personalization questionnaire. Factor-based calls were classified as disease and treatment coherence (DTC), self-administration (SA), treatment-related anxiety (TRA), and emotional burden (EB). The scores of 3–5 for TRA and EB and 1–3 for DTC and SA in the baseline personalization questionnaire were considered high risk, and the caregivers who had this score received an initial personalized factor-based telephone call (**Supplementary Material Tables S2**, **S3**). Subsequent biweekly telephone calls were scheduled over a 3-month period. The caregivers without high-risk baseline scores (low-risk group) did not receive any additional telephone calls. Two weeks after the final PSP telephone call, the caregivers were contacted by a nurse to complete a follow-up personalization questionnaire (**Supplementary Material Table S1**) to assess the changes in the questionnaire-based scores between the baseline and follow-up evaluations.

### 2.3 Statistical analysis

Data for all the participants were included in the analyses. Descriptive statistics were used to summarize the demographic data of the caregivers. The baseline and follow-up questionnaire data were compared to assess the questionnaire-based scoring pattern of the perceptions and beliefs of the caregivers. Normality data was examined to check assumptions for using parametric tests. Owing to the non-normal distribution of the sample, assumptions were not met, and hence, a nonparametric Wilcoxon signed-rank test was performed to compare changes in the questionnaire-based scores between the paired groups at baseline and follow-up evaluations. A *p-*value ≤0.05 was considered statistically significant.

## 3 Results

### 3.1 Risk factor characteristics

Of the caregivers in the high-risk group, 76 completed the entire program for the 3-month duration as well as the follow-up assessment. The patients in this sample group had a mean age of 10.9 (1.1–17.7) years, with 38.2% being female and 61.8% male. The mean age of the patients during treatment initiation was 8.7 years. The demographic characteristics of the sample group (patients of the high-risk worked) are presented in **Table 1A**. No differences were found between the characteristics of the patients of the sample group (n = 76) and total high-risk group (**Table 1B**). Demographic characteristics of the patients of the low-risk group are detailed in **Table 1C**. Furthermore, there were no differences between the low- and high-risk groups in terms of treatment duration or healthcare plans provided by the Health Maintenance Organization (*data not shown*).

**Table 1:** Demographic characteristics of the sample group (high risk worked), total high-risk group, and low-risk group.

Age (years)		Average (SD)		Median (min–max)	
A. Demographic characteristics of the sample group (high-risk worked group*)(n = 76)					
**Child’s age**		10.9 (3.3)		11.7 (1.1–17.7)	
**Age at treatment initiation**		8.7 (3.35)		9.3 (0.7–17.5)	
**Childs’s gender**		**n (%)**		
**Female**		29 (38.2)			
**Male**		47 (61.8)			
B. Demographic characteristics of the total high-risk group* (n = 162)					
**Child’s age**		10.5 (3.6)		11 (1.1–18.3)	
**Age at treatment initiation**		8.3 (3.5)		8.7 (0.4–17.5)	
**Child’s gender**		**n (%)**			
**Female**		69 (42.6)			
**Male**		93 (57.4)			
C. Demographic characteristics of the low-risk group* (n = 293)					
**Child’s age**		12.1 (3.2)		12.5 (2.8–18.3)	
**Age at treatment initiation**		9.7 (3.1)		9.9 (0.9–16.7)	
**Child’s gender**		**n (%)**		
**Female**		82 (28.0)			
**Male**		211 (72.0)			

*Group comprises the patients with GHD who were managed by the caregivers.

**SD,** standard deviation.

### 3.2 Caregivers’ perceptions and beliefs

**Figure 2:**
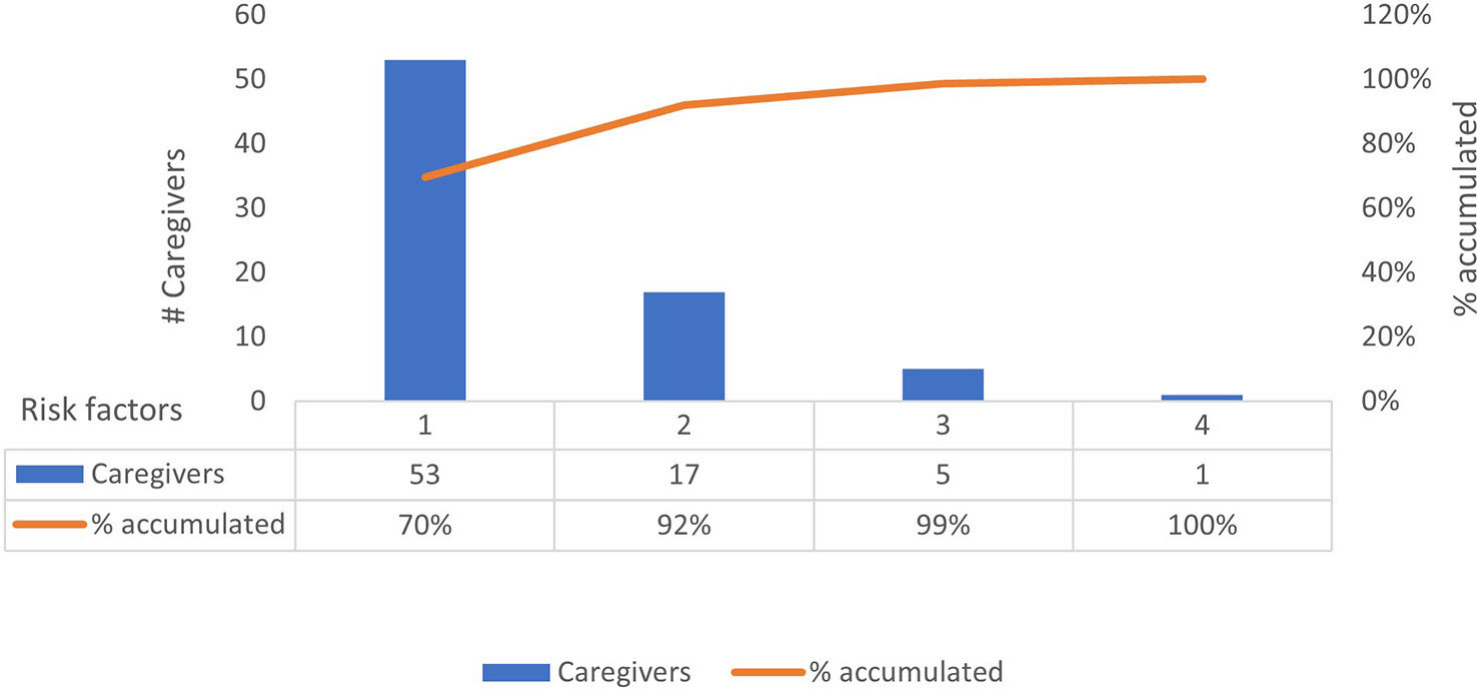
Scoring of risk factors by caregivers.

**Figure 3:**
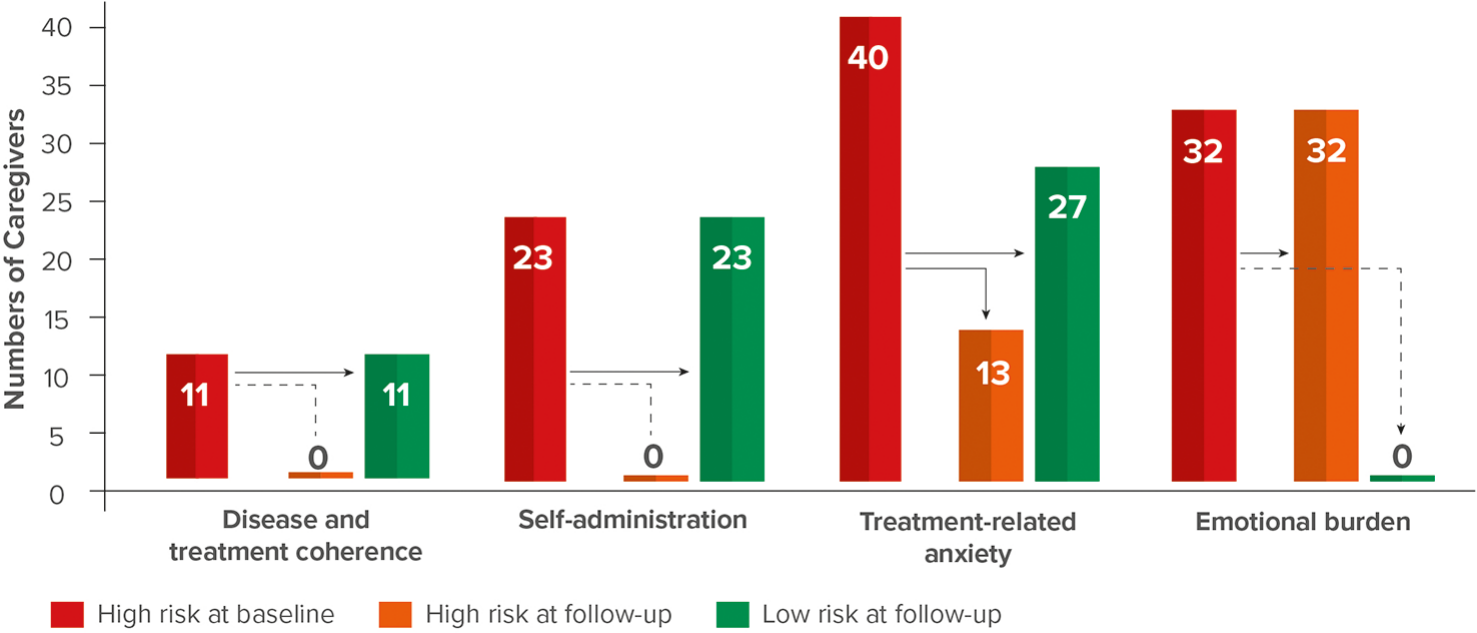
Change from high-risk to low-risk group following TUITEK^®^ PSP.

Statistically significant changes (*p* < 0.05) in the questionnaire scores of the baseline and follow-up evaluations were identified in the sample group across all four categories (**Table 2**). The proportion of caregivers with high-risk DTC and SA scores in the baseline questionnaire improved by 100%, with all the caregivers having low-risk scores DTC and SA scores at program completion. Furthermore, 67.5% of the caregivers with initially high-risk scores in the TRA category had low-risk scores after the 3-month observation period. Finally, while the overall EB scores of the caregivers in the high-risk group also significantly improved, no caregiver showed low-risk scores during or after the observation period (**Table 2**). The total number of categories for which individual caregivers provided high-risk scores were also quantified. We observed that 70% (n = 53) of the caregivers presented only one risk factor, 22% (n = 17) presented two risk factors, 7% (n = 5) presented three risk factors, and 1% (n = 1) presented four risk factors (**Figure 2**). The TUITEK^®^ PSP demonstrated that interventional support can lead to a positive shift in the perceptions and beliefs of caregivers. Most patients with GHD who were at high risk at baseline were considered low-risk after 3 months (**Figure 3**).

**Table 2:** Changes in caregiver scores between baseline and follow-up evaluations.

Patients in the high-risk category (n = 76)	Number of patients (n)	Baseline score (mean [SD]/median)	Follow-up score (mean [SD]/median)	Mean difference (*p*-value)*	Scoring	Indication of positive change
Disease and treatment coherence	11	2.45 (0.93)/3	4.00 (0.30)/4	1.55* (0.0025)	Direct	↑
Self-administration	23	2.17 (0.83)/2	4.00 (0.00)/4	1.83* (0.0000)	Direct	↑
Treatment-related anxiety	40	3.58 (0.75)/3	2.5 (0.91)/2	−1.08* (0.0000)	Reversed	↓
Emotional burden	32	3.69 (0.90)/3	3.13 (0.34)/3	−0.56* (0.0032)	Reversed	↓

*Wilcoxon signed-rank test. p-value <0.05; statistically significant.

SD, standard deviation.

## 4 Discussion

The appropriate use of r-hGH is essential for improving clinical health outcomes in children with GHD (1–3, 5, 6, 15, 16). This study aimed to determine the impact of the TUITEK^®^ PSP in Argentina. TUITEK^®^ is based on behavior change techniques and motivational interviewing (31–33). The program is designed to identify and address the beliefs and perceptions of caregivers regarding the condition and treatment of their child. Overall, our findings revealed that the TUITEK^®^ PSP can positively address and improve key disease- and treatment-related barriers, thereby aiding in the optimization of clinical outcomes and adherence among the caregivers of children who are prescribed r-hGH treatment in Argentina. The caregivers in this study exhibited improvements in their understanding of the disease and treatment, reduction in TRA and EB scores, and enhancement in confidence for the transition of parent-to-child administration (SA). Thus, these results demonstrate the program’s potential of supporting both patients with GHD and their caregivers to achieve a coherent understanding of their condition and treatment from the beginning of the treatment care pathway. At baseline, several caregivers reported experiencing anxiety regarding the r-hGH treatment, including fear of side effects and long-term implications or guilt surrounding injections. This behavior highlights a relevant burden of individuals caring for children with GHD. This notion has been evidenced by several studies that have reported that treatment anxiety is a continuous challenge among caregivers and a key factor associated with low treatment adherence (9, 21, 22, 34). Herein, treatment anxiety significantly reduced at follow-up, suggesting that tailored support constitutes an appropriate method for addressing the emotional needs of the caregivers. The survey data revealed that at baseline, many caregivers did not feel comfortable with their child taking over the responsibility of managing their own condition and self-administering their daily injections. As GHD is typically diagnosed in early childhood, usually, caregivers are initially responsible for their child’s treatment; hence, the transition of responsibility is often challenging for caregivers (19, 21, 35). However, this transition is crucial to ensure that the child establishes independent and effective self-management behaviors going forward. Our results demonstrate that the confidence of caregivers regarding this transition increased at follow-up, suggesting that the TUITEK^®^ PSP positively contributed to improving perceptions regarding patient SA.

Several studies have emphasized the impact of GHD on psychosocial functioning and quality of life of both patients and caregivers (19–22). In this respect, caregivers of children with GHD report enduring feelings of worry and concern about their child’s short- and long-term physical and emotional well-being. Herein, a strong EB of caring for a child with GHD was observed. It is well known that improving the perception of EB of caregivers can improve treatment outcomes, implying that the provision of emotional support to caregivers, for example, as part of the PSP, throughout the treatment journey is important. Notably, similar outcomes as those presented here were reported in a comparable study investigating the TUITEK^®^ PSP in Taiwan where improvements had been described across all four categories, indicating the versatility of the program among diverse cultural settings. As such, the TUITEK^®^ PSP offers a personalized intervention addressing the knowledge, beliefs, and perceptions of caregivers and positively impacting the treatment adherence in different geographies (30). In future, further analyses of the TUITEK^®^ PSP in other countries and r-hGH indications, including in Turner syndrome, CRF, and SGA, will provide evidence supporting the benefits of the program.

This study had some limitations. First, although the use of a small sample of caregivers (n = 76) challenged the generalizability of our findings, the presented findings are interim. Hence, further data from the ongoing PSP will provide a more comprehensive and robust set of findings. For this reason, our current results should be interpreted with a level of caution. Second, the nurse support calls, as part of the PSP, were delivered on a biweekly basis and limited to a follow-up of 3 months (30). Therefore, determining whether the changes observed within herein were sustained in the long term is out of the scope of this study. It is recommended that future investigations, where possible, should include longitudinal analyses, which would accordingly increase the strength of the findings. Furthermore, as adherence monitoring was beyond the scope of this evaluation, the impact of the TUITEK^®^ PSP on r-hGH adherence could not be established (30). As adherence is key to the success of r-hGH treatment and clinical outcomes among growth hormone disorders, it is recommended that this is addressed within future research.

## 5 Conclusions

Overall, the findings of this study indicate that the TUITEK^®^ PSP exerts a positive impact on the overall effectiveness of r-hGH treatment by identifying and addressing key adherence-related issues among caregivers of children with GHD and providing personalized adherence support. Moreover, the outcomes of this study were aligned with those observed when the TUITEK^®^ PSP was piloted in Taiwan, thus confirming the potential to exert a positive impact on adherence levels and self-management while supporting patients and their families, healthcare professionals, and the healthcare outlook.

## Data availability statement

The original contributions presented in the study are included in the article/**Supplementary Material**. Further inquiries can be directed to the corresponding author.

## Ethics statement

Ethical review and approval was not required for the study on human participants in accordance with the local legislation and institutional requirements. The patients/participants provided their written informed consent to participate in this study.

## Author contributions

AA, MC, MD, and JG contributed to the conception, design, and execution of the evaluation. SG wrote, drafted, and substantially revised the article. JR and AJ drafted the article and contributed to the acquisition, analysis, and interpretation of data. EK revised the articles critically for important intellectual content and agreed on all versions of the article and on the journal to which the article has been submitted. All authors agreed to be accountable for all aspects of the work. In addition, all authors approved the final manuscript.
