# Supplementary material for: Evaluating the TUITEK® patient support program in supporting caregivers of children diagnosed with growth hormone deficiency in Argentina
Source: Front Endocrinol (Lausanne). 2023 Apr 6;14:1129385. doi: 10.3389/fendo.2023.1129385 (PMC10117813; doi:10.3389/fendo.2023.1129385)
Supplement: Supplementary file 1 [file DataSheet_1.pdf]

Supplementary Material

1 Supplementary Tables

Table S1. Personalization questions and scoring

	Factors	Questions	Scoring	High risk cutoff
1	Disease and treatment coherence	How well do you feel you understand your child's treatment and condition?	1. Don't understand at all<br>2. Understand a little<br>3. Somewhat understand<br>4. Mostly understand<br>5. Understand very clearly	If the score is 1–3, the caregiver receives a disease and treatment coherence call.
2	Self-administration	How comfortable do you feel giving your child the responsibility of managing their condition and treatment?	1. Not at all<br>2. Slightly<br>3. Moderately<br>4. Very<br>5. Extremely	If the score is 1–3, the caregiver receives a self-administration call.
3	Treatment-related anxiety	How much does your child's treatment worry you? (E.g., do you feel worried about side effects or giving injections, if applicable)	1. Not at all<br>2. Slightly<br>3. Moderately<br>4. Very<br>5. Extremely	If the score is 3–5, the caregiver receives a treatment-related anxiety call.
4	Emotional burden	How much does your child's condition affect you emotionally? (E.g., does it make you angry, scared, upset, guilty, or frustrated?)	1. Not at all<br>2. Slightly<br>3. Moderately<br>4. Very<br>5. Extremely	If the score is 3–5, the caregiver receives an emotional burden call.

Table S2. Telephone call structure

Steps	Description
Greeting	The nurse will introduce/reintroduce themselves.
Follow-up	The nurse will follow up on the topic of the previous call (if applicable).
Explain the purpose of the call	The nurse will explain the purpose/objective of the call.
Explore and change	The nurse will probe to understand how the caregiver is impacted and how they are coping currently and signpost the caregiver to relevant resources based on their responses.
Set homework activity	The nurse will provide homework activity to encourage the caregiver to apply relevant strategies before the next call.
Closing	The nurse will summarize key points, ask the caregiver if they have any questions or concerns, and confirm the date and time for next appointment.

Table S3. Call guide outline

Factor-based call guide	Call objectives	Recommended interventional resources	Recommended behavioral change techniques
Disease and treatment coherence	To build and improve understanding of their condition.<br>To build and improve understanding of treatment.<br>To help caregivers understand the disease in the context of different experiences.<br>To manage expectations.<br>To promote adherence and monitoring of adherence.	GHD–The facts<br>Saizen–The facts<br>Managing Thinking Traps	Credible source<br>Information about health consequences<br>Monitoring of behavior by others with/without feedback<br>Framing/reframing<br>Reducing negative emotions
Self-administration	To support caregivers to develop their child's responsibility and resolve any challenges with administration.<br>To provide support for managing emotions surrounding "letting go" of treatment.	Seeking Support<br>Boosting Confidence<br>Making Life Easier Through Problem Solving	Social support<br>Focus on past success<br>Self-talk<br>Problem solving<br>Action planning
Treatment-related anxiety	To ensure sound understanding of Saizen.<br>To build confidence for administering Saizen.<br>To address any treatment-related concerns.	Saizen®–The Facts<br>Boosting Confidence<br>Managing Thinking Traps<br>Making Life Easier Through Problem Solving	Credible source<br>Information about health consequences<br>Monitoring of behavior by others with/without feedback<br>Framing/reframing<br>Reducing negative emotions<br>Focus on past success<br>Self-talk<br>Problem Solving

			Action Planning
Emotional burden	To build confidence for supporting the child with any emotional challenges.<br>To provide support for managing emotions, such as frustration, worry, and stress.<br>To build social support.	Seeking support<br>Managing Thinking Traps<br>Managing Stress<br>Managing Mood	Social support<br>Framing/reframing<br>Reducing negative emotions<br>Information about health consequences or about emotional consequences<br>Instructions on how to behave<br>Behavioral practice/rehearsal<br>Self-monitoring of outcomes of a behavior

GHD, growth hormone deficiency
